# Microfilament Depolymerization Is a Pre-requisite for Stem Cell Formation During *In vitro* Shoot Regeneration in *Arabidopsis*

**DOI:** 10.3389/fpls.2017.00158

**Published:** 2017-02-14

**Authors:** Li Ping Tang, Xiao Ming Li, Yu Xiu Dong, Xian Sheng Zhang, Ying Hua Su

**Affiliations:** ^1^State Key Laboratory of Crop Biology, College of Life Sciences, Shandong Agricultural UniversityTaian, China; ^2^Shandong Provincial Key Laboratory of Agricultural Microbiology, College of Plant Protection, Shandong Agricultural UniversityTaian, China

**Keywords:** microfilament depolymerization, stem cell formation, auxin distribution, auxin polar transport, shoot regeneration, *Arabidopsis*

## Abstract

*De novo* shoot regeneration is widely used in fundamental studies and agricultural applications. Actin microfilaments are involved in many aspects of plant cell division, cell morphogenesis and cell signal transduction. However, the function of actin microfilaments during *de novo* shoot regeneration is poorly understood. Here, we investigated the organization of actin microfilaments during this process and found that stem cell formation was associated with microfilament depolymerization. Furthermore, inhibition of microfilament depolymerization by phalloidin treatment or downregulation of actin depolymerizing factors (ADFs) restrained stem cell initiation and shoot regeneration. Inhibition of *ADF* expression affected the architecture of microfilaments during stem cell formation, and the polar transport and distribution of auxin were also disrupted. Together, our results demonstrate that organization of the microfilament cytoskeleton play important roles in stem cell formation and shoot meristem induction during shoot regeneration.

## Introduction

Plant somatic cells can be reprogrammed to generate various organs under defined physical and chemical conditions, a process called *de novo* organogenesis. This phenomenon is not only critical for *in vitro* plant propagation and biotechnology, but also useful for understanding plant developmental regulatory mechanisms (Sugiyama, 2000). The patterns of plant *de novo* organogenesis depend on the specific balance of applied exogenous hormones. High ratios of auxin/cytokinin lead to root regeneration, while high cytokinin/auxin ratios induce shoot regeneration. With high concentrations of both auxin and cytokinin, callus can be generated (Skoog and Miller, 1957; Bhojwani and Razdan, 1996; Che et al., 2002).

A number of studies have been performed to understand the molecular mechanisms underlying *de novo* shoot regeneration in *Arabidopsis* (Gordon et al., 2007; Li et al., 2011; Cheng et al., 2013). Auxin is the best-known hormone exhibiting local accumulation and responses during shoot regeneration. Dynamic distribution patterns of the auxin response were clearly shown in the *in vitro* shoot initiation (Gordon et al., 2007). During callus formation on auxin-rich callus induction medium (CIM), auxin responsive signals represented by the *DR5rev* signals are uniformly present in clusters of proliferating callus cells at the edges of the callus. After transfer to cytokinin-rich shoot induction medium (SIM), low auxin-responsive signals are required for initiation of the shoot apical meristem (SAM) in the callus. *WUSCHEL* (*WUS*), a transcription factor, plays a key role in *de novo* shoot regeneration (Gallois et al., 2004; Gordon et al., 2007). WUS is required for shoot stem-cell formation and maintenance in SAM, on which many signaling pathways converge (Dodsworth, 2009). During *de novo* shoot regeneration in *Arabidopsis*, WUS is upregulated in the center of the regenerated SAM, which is required for stem cell induction and subsequent shoot formation (Gordon et al., 2007; Cheng et al., 2013). WUS promotes the initiation and maintenance of the overlying stem cells by stimulating the activity of *CLAVATA3* (*CLV3*) therein, which is a small secreted peptide as a stem cell marker (Mayer et al., 1998; Fletcher et al., 1999; Brand et al., 2000). Induction of the WUS expression during shoot regeneration was regulated by the master phytohormone auxin (Cheng et al., 2013). Auxin polar transport mediated by the PINFORMED efflux carrier proteins (PINs) contributes to the initiation and maintenance of auxin-responsive gradients in specific callus tissues during shoot regeneration (Cheng et al., 2013). Polarized membrane localization of PIN1 at initiation sites of *de novo* SAMs can be induced by SIM incubation, which contributes to the spatially distributed auxin response. Shoot regeneration is severely reduced in the plants expressing antisense *PIN1*. Application of the auxin transport inhibitor naphthylphthalamic acid (NPA) disrupts the spatiotemporal auxin response and shoots regeneration, demonstrating that auxin polar transport and the asymmetric distribution of the auxin response are required for initiation of SAMs during shoot regeneration (Cheng et al., 2013).

The microfilament cytoskeleton, a major component of the plant cell cytoskeleton, is involved in many aspects of cell division, cell morphogenesis, and the establishment and maintenance of cell polarity by filament polymerization and depolymerization (Vantard and Blanchoin, 2002). Interestingly, the polar transport and distribution of auxin play critical roles in regulating cytoskeletal organization, which is important for cell polarization and morphogenesis (Pan et al., 2015; Wu et al., 2015). Proteins involved in the actin organization and dynamics have been characterized in *Arabidopsis* roots (Takáč et al., 2017). As an actin binding protein, actin depolymerizing factor (ADF) controls the actin organization and regulates various plant cell development and morphogenesis processes (Jiang et al., 1997; Dong et al., 2001; Allwood et al., 2002; Henty-Ridilla et al., 2014). Previous studies have demonstrated that *ADF9* is expressed in the SAM and controls multicellular development including callus formation generated by root explants *via* both cytoplasmic and nuclear processes in *Arabidopsis* (Burgos-Rivera et al., 2008). Furthermore, *ADF4* plays a role in pathogen perception, defense activation and transcription through the regulation of actin cytoskeletal dynamics and *R-gene* transcription (Porter et al., 2012). In addition, the actin cytoskeleton and its dynamics have roles in responses to abiotic and biotic stimuli.

During *in vitro* shoot regeneration in *Arabidopsis*, a group of cells in callus can develop into organizing center cells through transdifferentiation (Gordon et al., 2007; Cheng et al., 2013). It has been reported that the coexpression of ACT7 with other actin proteins is required for normal callus formation (Kandasamy et al., 2001). However, little is known about the organization of the microfilament cytoskeleton of callus cells during this process. In this study, we showed that stem cell formation in callus is associated with the process of microfilament depolymerization. Inhibition of microfilament depolymerization by phalloidin treatment or downregulation of *ADF*s inhibited stem cell formation and shoot induction. Furthermore, repression of *ADF* expression disrupted the polar transport and distribution of auxin in callus. Depolymerization of the microfilament cytoskeleton is thus required for stem cell formation during shoot regeneration, through mediating the polar transport and distribution of auxin.

## Materials and Methods

### Plant Materials

*Arabidopsis thaliana* plants used in this study were of the Columbia (Col) and Landsberg *erecta* (L*er*) ecotypes. The origins and ecotypes of the transgenic lines and mutants were as follows: *pWUS::DsRed-N7* (Ler; Gordon et al., 2007), *pCLV3::GFP-ER* reporter lines (Ler; Lenhard and Laux, 2003), *pWUS::GUS* and *pCLV3::GUS* (Col; Su et al., 2009), *DR5rev::GFP* and *pPIN1::PIN1-GFP* (Col; Xu et al., 2006), *35S::GFP-ABD2-GFP* (Col; Wang et al., 2008), *CYCB1;1::GUS* (Col; Colón-Carmona et al., 1999). The *pWUS::DsRED-N7* plants were crossed with *35S::GFP-ABD2-GFP* plants. The estradiol-inducible XVE binary vector (Zuo et al., 2000) was kindly provided by Dr. Nam-Hai Chua (Rockefeller University, New York, NY, USA).

### Plant Growth Conditions and Shoot Regeneration

*Arabidopsis* seeds were sterilized and plated on germination medium (Murashige and Skoog, 1962). The plates were kept at 4°C for 2 days to overcome dormancy, and then transferred to a culture room at 22°C with a 16-h-light/8-h-dark cycle for 7 days. Young seedlings were transplanted into vermiculite and grown under the conditions as described above until harvesting of siliques.

*De novo* shoot regeneration was performed as described by Li et al. (2011). After cold treatment, the seeds were vertically cultivated in the plate under sterile conditions (light intensity of 40 μmol photons m^-2^ s^-1^, 22°C, under a 16-h-light/8-h-dark cycle) for approximately 7 days, and root explants of 5 mm length were excised from the seedlings. Then, the explants were transferred onto CIM consisting of Gamborg’s B5 medium (Gamborg et al., 1968) containing 2% glucose, 0.5 g/L MES, 0.2 μmol/L kinetin, and 2.2 μmol/L 2,4-dichlorophenoxyacetic acid (2,4-D) with 0.8% agar, and incubated for 6 days to induce callus production. Finally, the calli were transferred onto SIM with 2% glucose, 0.5 g/L MES, 0.9 μmol/L 3-indoleacetic acid, and 0.5 μmol/L 2-isopentenyladenine to induce shoot regeneration.

### Actin Staining in Callus

Actin filaments in callus were stained with Alexa-488 phalloidin as previously described (Zheng et al., 2013). To observe the organization of the actin cytoskeleton during stem cell formation in callus of shoot regeneration, callus from *pWUS::DsRed-N7* plants were subsequently subjected to fixation and staining with Alexa-488 phalloidin. Actin filaments in the callus were observed with a confocal laser scanning microscope (Leica TCS SP5, Germany) equipped with a 40× oil objective. The fluorescent phalloidin was excited with the 488-nm line of an argon laser.

### Construction of an Artificial MicroRNA (amiRNA) Vector

The artificial microRNA (amiRNA) silencing procedure was performed as described by Schwab et al. (2006). The WMD3^1^ (Web MicroRNA Designer3) software was used to design specific primers for *ADF1* (AT3G46010), *ADF2* (AT3G46000), *ADF3* (AT5G59880), and *ADF4* (AT5G59890). The plasmid pRS300 containing the endogenous *Arabidopsis* MIR319a precursor (Schwab et al., 2006) was used as a template, and ath-MIR319a was replaced by overlapping PCR with the primers A, B and adf1 adf2 adf3 adf4 I–IV (**Supplementary Table S1**). Then, the amiRNA cloned into the plasmid was enzymatically digested and ligated into the *pER8* expression vector, which was then transformed into plants through *Agrobacterium*-mediated floral dip method. Hygromycin was used to screen positive transgenic lines.

### Total RNA Extraction and Quantitative Real-Time PCR (qRT–PCR)

Total RNAs were isolated from various calli using Trizol reagent (Invitrogen). The qRT-PCR reactions were performed for each cDNA dilution using SYBR Green Master mix with Chromo4 according to the manufacturer’s protocol (Bio-Rad). All primers used for qRT-PCR are shown in **Supplementary Table S1**. The relative expression level of each gene was standardized to that of the housekeeping gene *TUBULIN2*, and all measurements were carried out in three biological replicates. The results were analyzed using the comparative *C*_T_ method, and means and standard deviations were calculated.

### β-GUS Assays

To investigate the expression patterns of *pWUS::GUS* during shoot initiation, a GUS histochemical staining assay was performed following Cheng et al. (2014). Then, the materials were destained with 70% ethanol for imaging.

### Chemicals and Induction

To determine the effect of phalloidin on shoot induction, calli were cultured on SIM containing various concentrations of phalloidin (prepared in DMSO as a 20 mmol/L stock; Sigma) for 20 days. The regeneration frequencies of shoots under treatments of various concentrations of phalloidin were statistically analyzed, and 90 samples for each treatment were collected. Means and standard deviations were calculated (shown as ‘mean ± SD’ in **Table 1**). Callus cultured on SIM containing 5 μmol/L phalloidin for 0, 4, 8, 12, and 16 days were individually harvested to isolate total RNA for qRT-PCR analysis.

**Table 1:** Regeneration frequencies of shoots with treatment of different concentrations of phalloidin.

	Mock	100 nmol/L phalloidin	1 μmol/L phalloidin	5 μmol/L phalloidin
percentage^i^	90.85%^a^	78.35%^b^	49.99%^c^	10.05%^d^
number^ii^	15 ± 5^a^	12 ± 6^ab^	6 ± 4^b^	4 ± 2^c^


*^i^Proportion of explants that could regenerate shoots.*

*^ii^The number of shoots regenerated from each callus (mean ± SE, *n* = 90). Calli were induced on SIM for 20 days.*

*^a-d^Different superscripts are significantly different by ANOVA test, *P* < 0.01.*

To induce transcription of artificial microRNAs, the calli were cultured on SIM with 10 μmol/L estradiol (prepared in DMSO as a 10 mmol/L stock; Sigma) for 20 days and estradiol was added every 2 days. Total RNA was isolated from the callus on SIM with estradiol at 12 days and used for qRT-PCR to determine the expression of *ADF* genes.

### Imaging Conditions

An Olympus JM dissecting microscope was used to photograph the callus morphologies. To investigate the green fluorescence images for actin staining and live cell imaging, samples were observed using a Leica TCS SP5 confocal microscope with a 40× oil lens. For each stage, at least 40 samples were imaged to confirm the structure of actin. To investigate red and green fluorescence images for co-localization between WUS and ABD2 in callus cells and expression patterns of WUS, CLV3, PIN1, and DR5 reporter lines, samples were observed using a 40× oil lens. For each gene marker line at various stages, at least 30 samples were imaged to confirm the expression pattern of a particular marker at each stage. The specific sets of filters used for each marker were similar to those described by Heisler et al. (2005). Leica LAS AF Lite software was used for confocal images processing.

### Quantitative Image Analysis of ABD2-GFP Reporter and Actin Staining Fluorescence Density and Skewness of the Actin Filaments in Callus Cells

The density and skewness of actin filaments were quantified by ImageJ according to Higaki et al. (2010). To estimate actin filaments density, we defined the occupancy of the fluorescence signal as calculated from the skeletonized image of the callus cells. The occupancy becomes lower as the actin filaments are depolymerized or fragmented. To estimate actin filaments polymerization and bundling, we used the skewness of the intensity distribution of the microfilament pixels as an indicator of its polymerization and bundling. The skewness decreases as the fluorescence intensity in the pixels decreases as a result of actin filaments depolymerized or fragmented. Individual cells were segmented manually and actin filaments at the cell border were eliminated. For ABD2 reporter lines and actin staining samples at each developmental stage, more than 50 callus cells were used for the analysis.

## Results

### Stem Cell Initiation is Associated with the Process of Microfilament Depolymerization During *In vitro* Shoot Regeneration

**FIGURE 1:**
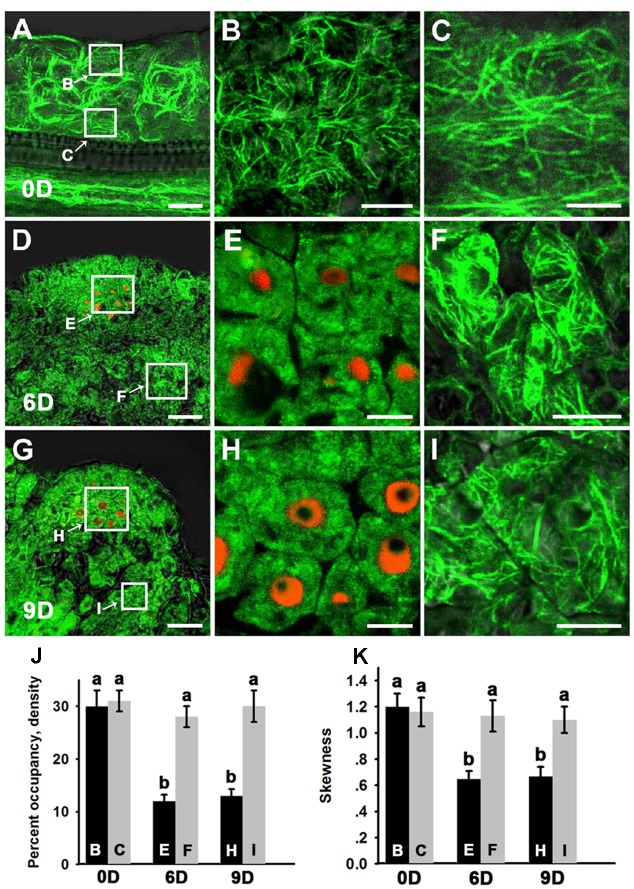
**Organization of actin filaments during shoot regeneration.** **(A)** Callus cultured on SIM for 0 days. **(B,C)** Magnification of the areas indicated by the arrows in **(A)**. Strong green signals of polymerized and bundled filaments were detected both in the epidermal callus cells **(B)** and in the inner layers of callus cells close to the vascular tissue **(C)**. **(D)** Callus cultured on SIM for 6 days. **(E,F)** Magnification of the areas indicated by the arrows in **(D)**. Microfilaments became more fragmented and less bundled in the *WUS*-expressing cells **(E)** compared with the callus cells surrounding these *WUS*-expressing cells **(F)**. **(G)** Callus cultured on SIM for 9 days. **(H,I)** Magnification of the areas indicated by the arrows in **(G)**. The green signals of microfilaments mainly showed fragmented distributions in the *WUS*-expressing organizing center cells of the *de novo* shoot meristem **(H)** compared with the callus cells close to the shoot primordial **(I)**. Green signal represents the fluorescence of *35S::GFP-ABD2-GFP*, red signal represents the fluorescence of *pWUS::DsRED-N7*. Bars = 10 μm. **(J)** The average filament density measured in the callus cells shown in **(B,C,E**,**F,H,I)**. The *WUS*-expressing cells had a lower density value than the callus cells. **(K)** The extent of filament bundling (skewness) measured in the callus cells shown in **(B,C,E,F,H,I)**. The *WUS*-expressing cells had a lower skewness value than the callus cells. Different lowercases in **(J,K)** are significantly different by ANOVA test, *P* < 0.01. Error bars represent standard deviations from triplicate measurements.

A *de novo* shoot regeneration system was established using root explants in *Arabidopsis*. In a two-step regeneration process, the explants were cultured on an auxin-rich CIM for 6 days and then incubated on a cytokinin-rich SIM to induce shoot regeneration (Gordon et al., 2007). Stem cell initiation and SAM formation is developmentally induced during the shoot regeneration process, and the expression of *WUS* is the earliest event to mark stem cell initiation (Gordon et al., 2007; Cheng et al., 2013). Fimbrin actin binding domain 2 (ABD2)-based filamentous actin (F-actin) reporters have emerged as powerful tools to study microfilament organization in living plants. To observe the organization of the microfilament cytoskeleton in stem cell initiation during shoot regeneration, *pWUS::DsRED-N7/35S::GFP-ABD2-GFP* double reporter lines were used to obtain explants. After incubation on CIM for 6 days (SIM for 0 day), neither *WUS* expression nor stem cell initiation was induced in the callus (**Figure 1A**). Strong GFP signals of polymerized and bundled filaments were detected both in the epidermal callus cells (**Figures 1A,B**) and in the inner layers of callus cells close to the vascular tissue (**Figures 1A,C**), suggesting that the microfilaments were mainly polymerized and bundled in these callus cells. However, regional expression of the *WUS* gene was detected in callus cultured on SIM for 6 days, implying stem cells were initiated (**Figure 1D**). At this time, microfilaments became fragmented in the *WUS*-expressing cells (**Figures 1D,E**), and were less bundled compared with the callus cells surrounding these *WUS*-expressing cells (**Figures 1D,F**). After the callus was cultured on SIM for 9 days, shoot primordia were formed (**Figure 1G**). The microfilaments mainly showed fragmented distributions in the *WUS*-expressing organizing center cells of the *de novo* shoot meristem (**Figures 1G,H**). However, the microfilaments in the callus cells close to the shoot primordia were still polymerized and bundled filaments (**Figures 1G,I**). To quantitatively analyze the actin architecture, we measured the skewness and density (Higaki et al., 2010) parameters to determine the extent of actin filament bundling and the percentage of occupancy of actin filaments in the *WUS*-expressing cells and callus cells. Consequently, the *WUS*-expressing cells had a lower density value than the callus cells (**Figure 1J**). Moreover, the actin filaments were bundled with higher skewness in the callus cells than the *WUS*-expressing cells (**Figure 1K**). However, the density and skewness values showed no significant difference in callus cells cultured on SIM for 6 days and 9 days compared with those cultured on SIM for 0 day (**Figures 1J,K**). We further confirmed the actin architecture observations in the stem cell initiation process using fixation and staining of the microfilament cytoskeleton. Callus derived from *pWUS::DsRED-N7* plants were subjected to F-actin staining with Alexa-488 phalloidin. Similar to the ABD2 marker used to visualize actin architecture, the fluorescence signals associated with F-actin staining by Alexa-488 phalloidin were weaker in *WUS*-expressing cells compared with the callus cells (**Supplementary Figure S1**). The density and skewness values were much lower in *WUS*-expressing cells. These observations indicate that stem cell initiation in the induced callus is associated with the depolymerization and fragmentation of the microfilament cytoskeleton during *in vitro* shoot regeneration.

### Effects of Microfilament Cytoskeleton Organization on Stem Cell Formation and Shoot Regeneration

**FIGURE 2:**
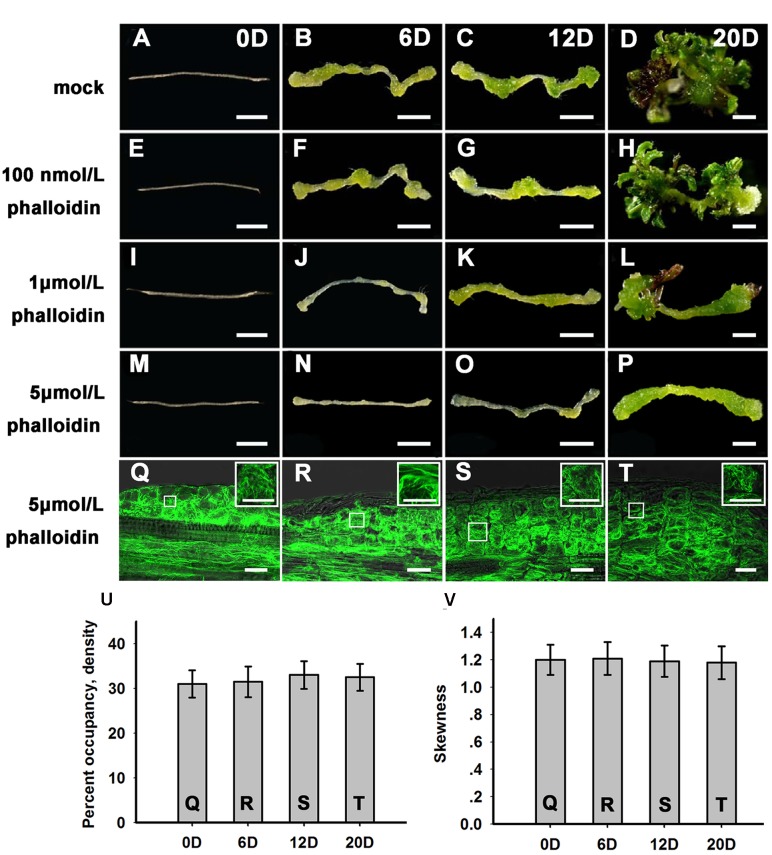
**Shoot regeneration under treatment with different concentrations of phalloidin.** **(A–D)** Shoot regeneration of wild type plants when cultured on SIM with DMSO for 0, 6, 12, and 20 days. **(E–H)** Shoot regeneration of wild type plants when cultured on SIM containing 100 nmol/L phalloidin (prepared in DMSO) for 0, 6, 12, and 20 days, which had minor effects on the induction of shoots. **(I–L)** Shoot regeneration of wild type plants when cultured on SIM containing 1 μmol/L phalloidin for 0, 6, 12, and 20 days, which caused remarkable decreases in the production of shoots. **(M–P)** Shoot regeneration of wild type plants when cultured on SIM containing 5 μmol/L phalloidin for 0, 6, 12, and 20 days, which almost completely inhibited shoot regeneration. Bars = 1 mm. **(Q–T)** *35S::GFP-ABD2-GFP* signals in callus when cultured on SIM containing 5 μmol/L phalloidin for 0, 6, 12, and 20 days. The actin filaments were still polymerized and bundled with a filamentous distribution in these callus cells. Bars = 20 μm. **(U)** The average filament density measured in the callus cells shown in **(Q,R–T)**. **(V)** The extent of filament bundling (skewness) measured in the callus cells shown in **(Q,R–T)**. No overt differences were observed in the density and skewness values of these cells. Error bars represent standard deviations from triplicate measurements.

To further examine the function of microfilament cytoskeleton organization during shoot regeneration, phalloidin, an inhibitor of microfilament depolymerization, was added to the SIM at different concentrations. As shown in **Figures 2A–H** and **Table 1**, 100 nmol/L phalloidin had minor effects on the induction of shoots. A high concentration of 1 μmol/L phalloidin caused remarkable decreases in the production of shoots with severe abnormal morphologies (**Figures 2I–L**; **Table 1**). Treatment with 5 μmol/L phalloidin almost completely inhibited shoot regeneration (**Figures 2M–P**; **Table 1**). In order to assess the status of cell growth and division during the phalloidin experiments, the fresh weight of callus was measured (**Supplementary Figure S2A**). Even treated with 5 μmol/L phalloidin, the callus continually increased their fresh weight during shoot induction on SIM. The *Arabidopsis* mitotic cyclin *CYCB1;1* is excellent marker for cells undergoing mitosis, which is expressed around the G2/M transition (Colón-Carmona et al., 1999). The expression of *CYCB1;1::GUS* was retained in the callus cells under treatment of 5 μmol/L phalloidin (**Supplementary Figures S2C,D**). These results eliminated the general impairment of cell growth and division during phalloidin treatment. We further detected the organization of the actin filaments in the callus cells on SIM treated with 5 μmol/L phalloidin. The actin filaments were still polymerized and bundled with a filamentous distribution in these callus cells (**Figures 2Q–T**), and no overt differences were observed in the density and skewness values (**Figures 2U,V**). These results indicated that phalloidin treatment leading to inhibition of microfilament depolymerization in the callus cells inhibited shoot induction.

**FIGURE 3:**
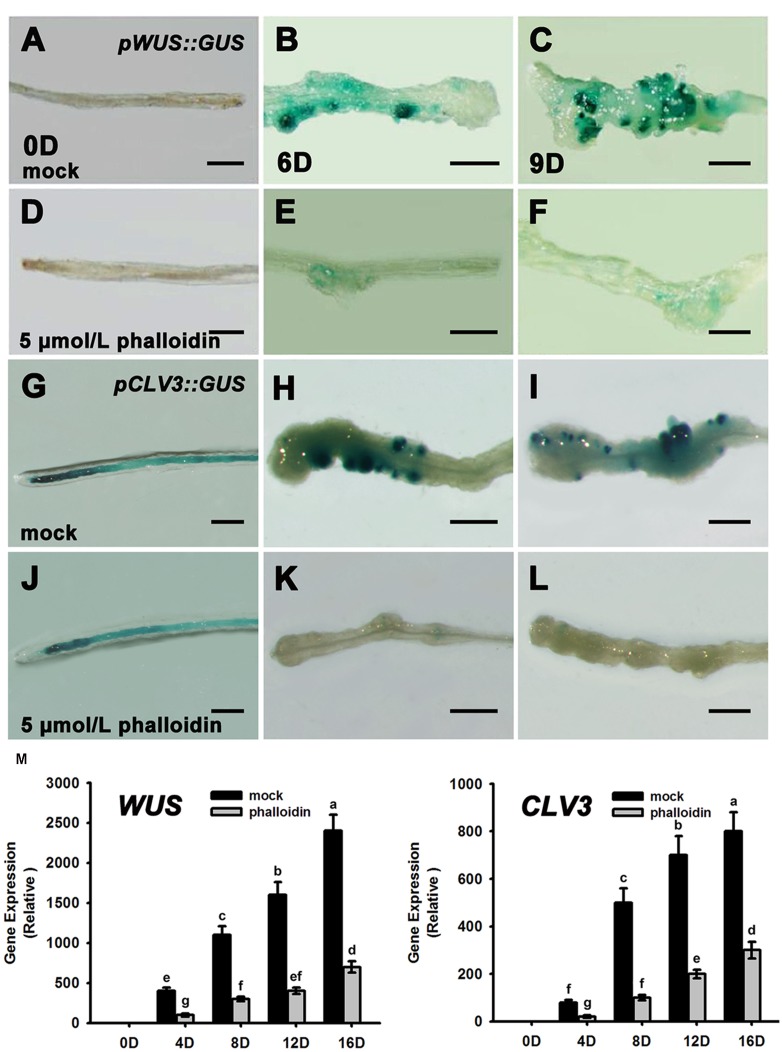
**Expression patterns of *WUS* and *CLV3* in calli treated with phalloidin.** **(A–C)** Expression patterns of *pWUS::GUS* in callus cultured on SIM with DMSO for 0, 6, and 9 days. **(D–F)** Expression patterns of *pWUS::GUS* in callus cultured on SIM containing 5 μmol/L phalloidin for 0, 6, and 9 days. *WUS* expression was not detected in callus**. (G–I)** Expression patterns of *pCLV3::GUS* in callus cultured on SIM with DMSO for 0, 6, and 9 days. **(J–L)** Expression patterns of *pCLV3::GUS* in callus cultured on SIM containing 5 μmol/L phalloidin for 0, 6, and 9 days. *CLV3* expression was not detected in callus. Bars = 1 mm. **(M)** Relative expression levels of *WUS* and *CLV3* in calli as determined by qRT-PCR. The transcript levels of both *WUS* and *CLV3* were reduced in callus treated with phalloidin. Mock, callus cultured on SIM with DMSO; phalloidin, callus cultured on SIM containing 5 μmol/L phalloidin. Different lowercases are significantly different by ANOVA test, *P* < 0.01. Error bars represent standard deviations from triplicate measurements.

Previous studies have reported that *WUS* and *CLV3* expression marks stem cell formation in shoot meristem (Laux et al., 1996; Mayer et al., 1998; Schoof et al., 2000; Weigel and Jürgens, 2002). WUS-expressing cells in the organizing center establish and maintain stem cell populations markerd by *CLV3* expression within the central zone of the shoot meristem. Therefore, we studied the expression patterns of *WUS* and *CLV3* during shoot meristem induction under phalloidin treatment using *pWUS::GUS* and *pCLV3::GUS* reporter lines. As shown in **Figures 3A,B**, *WUS* transcription was induced in several restricted regions of the callus grown on SIM without phalloidin for 6 days. Subsequently, shoot primordia appeared in these *WUS*-expressing regions and the *WUS* signals were maintained in the organizing center cells of the shoot primordia (**Figure 3C**). By contrast, *WUS* expression was not detected in callus after 9 days of culture on SIM with 5 μmol/L phalloidin (**Figures 3D–F**). Consequently, the suppression of *WUS* expression was accompanied by inhibition of shoot formation in the phalloidin-treated callus. In shoot regeneration, the induction of *WUS* expression specifies stem cells that are marked by *CLV3* expression in callus (Cheng et al., 2010). Similar to *WUS. CLV3* expression was either not detected in callus on SIM treated with 5 μmol/L phalloidin (**Figures 3G–L**). The expression patterns of *WUS* and *CLV3* under 5 μmol/L phalloidin treatment were further determined by qRT-PCR analysis. As expected, the transcript levels of both *WUS* and *CLV3* were reduced in callus treated with phalloidin compared with those in untreated tissues within 16 days culture on SIM (**Figure 3M**), suggesting that inhibition of microfilament depolymerization in callus cells might disturb shoot regeneration by suppressing the expression of *WUS* and *CLV3* genes.

### Downregulation of *ADF*s Reduced the Rates and Frequencies of Shoot Regeneration

**FIGURE 4:**
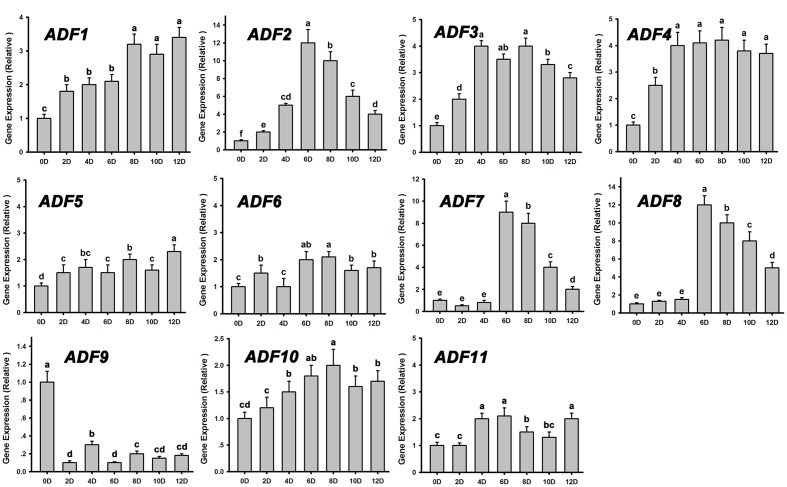
**Relative expression levels of *ADF*s during shoot regeneration.** Different lowercases are significantly different by ANOVA test, *P* < 0.01. Error bars represent standard deviations from triplicate measurements.

Actin depolymerizing factors (ADFs) are a kind of actin-binding proteins of low molecular weight that exist widely in eukaryotes and play important roles in microfilament depolymerization and polymerization (Andrianantoandro and Pollard, 2006; Pei et al., 2012). We analyzed the expression patterns of *ADFs* during *de novo* shoot regeneration after cultured on SIM for 12 days using RT-PCR. Most of the *ADF* genes were upregulated at the early stages of shoot induction, especially *ADF1. ADF2. ADF3*, and *ADF4*, while the actin polymerizing factor *ADF9* was significantly downregulated (**Figure 4**). These results were consistent with the depolymerization of microfilaments during shoot meristem formation.

**FIGURE 5:**
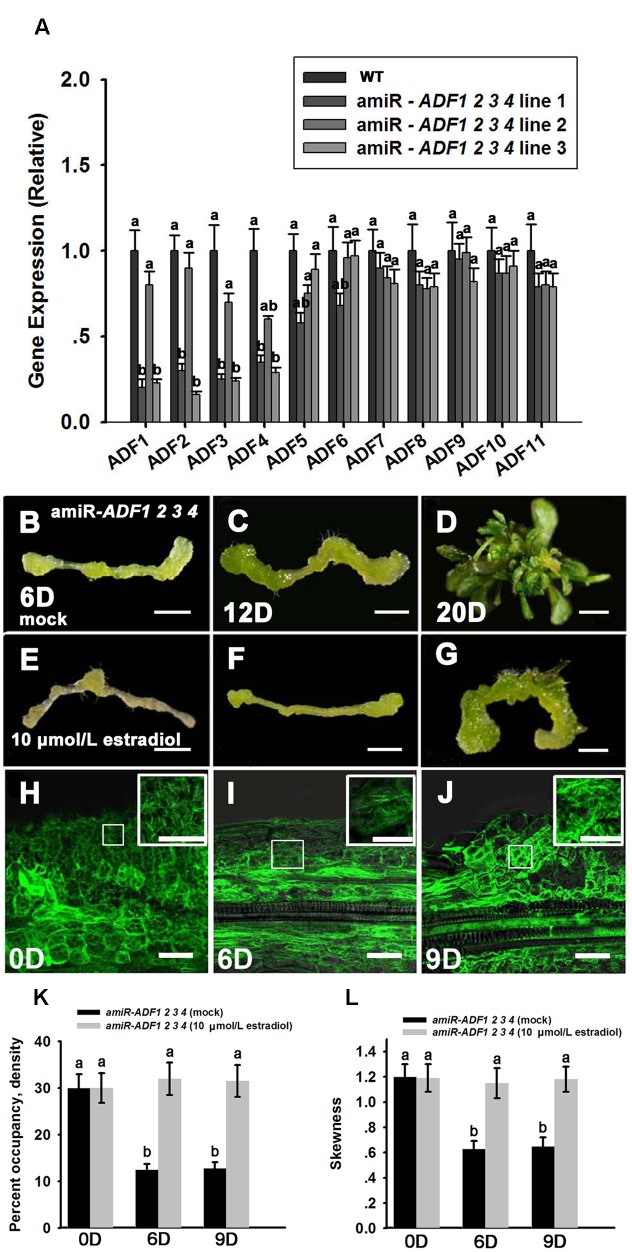
**Shoot regeneration from amiR-*ADF1 2 3 4* transgenic lines induced by estradiol.** **(A)** Relative expression levels of *ADF*s in wild type (WT) and estradiol-induced different transgenic lines by qRT-PCR analysis. The transcript levels of *ADF1. ADF2. ADF3*, and *ADF4* were downregulated most obviously in transgenic line 3. Different lowercases are significantly different by ANOVA test, *P* < 0.01. Error bars represent standard deviations from triplicate measurements. **(B–D)** Shoot regeneration of non-induced transgenic plants when cultured on SIM without estradiol for 6, 12, and 20 days. **(E–G)** Shoot regeneration of induced transgenic plants when cultured on SIM containing 10 μmol/L estradiol for 6, 12, and 20 days. Few shoot primordia emerged from the estradiol-induced transgenic plants. Bars = 1 mm. **(H–J)** *35S::GFP-ABD2-GFP* signals in callus from induced transgenic plants when cultured on SIM containing 10 μmol/L estradiol for 0, 6, and 9 days. The actin filaments were still polymerized and bundled with a filamentous distribution in the callus cells. Bars = 20 μm. **(K)** The average filament density measured in the stem cells under mock control and callus cells shown in **(H–J)**. **(L)** The extent of filament bundling (skewness) measured in the stem cells under mock control and callus cells shown in **(H–J)**. No overt differences were observed in the density and skewness values in the callus cells cultured with estradiol. Different lowercases are significantly different by ANOVA test, *P* < 0.01. Error bars represent standard deviations from triplicate measurements.

The *adf* single mutant did not show obvious phenotypes during shoot regeneration (data not shown), implying that the *ADF* family genes were functionally redundant. *ADF* subclass I, including *ADF1. ADF2. ADF3* and *ADF4*, is constitutively and highly expressed in various reproductive and vegetative organs except pollen tubes (Ruzicka et al., 2007). Here, the artificial microRNA technology was used to knock down these four *ADF* genes simultaneously. Three lines of T_2_ generation plants (amiR-*ADF1 2 3 4*) were selected to analyze the expression levels of the *ADF*s. As shown in **Figure 5A**, *ADF1. ADF2. ADF3*, and *ADF4* were all significantly downregulated in the three estradiol-induced transgenic lines, while the expression of the other *ADF* genes was almost the same as in the non-induced transgenic plants. As the transcript levels of *ADF1. ADF2. ADF3*, and *ADF4* were downregulated most obviously in transgenic line 3, we selected this line for further analysis.

To analyze shoot regeneration in the transgenic plants, estradiol was added to the SIM to induce the expression of the artificial microRNA that inhibited *ADF1. ADF2. ADF3*, and *ADF4* expressions. Several green shoot primordia regenerated from non-induced transgenic plants were observed after growth on SIM for 12 days, and numerous shoots were regenerated after 20 days in the SIM (**Figures 5B–D**; **Table 2**). By contrast, few shoot primordia emerged from the estradiol-induced transgenic plants grown on SIM for 12 days (**Figures 5E,F**). Even if the induced callus cultured for 20 days, the number of shoots was much less than that in non-induced callus (**Figure 5G**; **Table 2**). Thus, we confirmed that simultaneously suppressing the expression of *ADF1. ADF2. ADF3*, and *ADF4* could reduce the rates and frequencies of shoot regeneration. Like the wild type plants, the callus of the amiR-*ADF1 2 3 4* transgenic plants still increased their fresh weight during shoot induction on SIM, eliminating the general impairment of cell growth and division (**Supplementary Figure S2B**). To further analyze the organization of the microfilament cytoskeleton in the amiR-*ADF1 2 3 4* transgenic plants during shoot regeneration, *35S::GFP-ABD2-GFP* was transformed into the amiR-*ADF1 2 3 4* transgenic lines. As shown in **Figures 5H–J**, the actin filaments were still polymerized and bundled with a filamentous distribution in the callus cells of amiR-*ADF1 2 3 4* plants cultured on SIM with estradiol for 9 days. Furthermore, no overt differences were observed in the density and skewness values (**Figures 5K,L**). These results suggest that inhibition of *ADF1. ADF2. ADF3*, and *ADF4* functions can inhibit the normal depolymerization of microfilaments, resulting in the repression of shoot regeneration.

**Table 2:** Shoots regeneration frequencies of amiR-*ADF1 2 3 4* transgenic plants in col ecotype.

	Non-induced transgenic plant	Induced transgenic plant
Percentage^a^	86.70%	35.04% (^∗∗∗c^)
Number^b^	12 ± 3	6 ± 2 (^∗^)


*^a^Proportion of explants that could regenerate shoots.*

*^b^The number of shoots regenerated from each callus (mean ± SE, *n* = 120). Calli were induced on SIM for 20 days.*

*^c^Significant difference analysis: ^∗∗∗^*P* < 0.001; ^∗∗^*P* < 0.01; ^∗^*P* < 0.05.*

**FIGURE 6:**
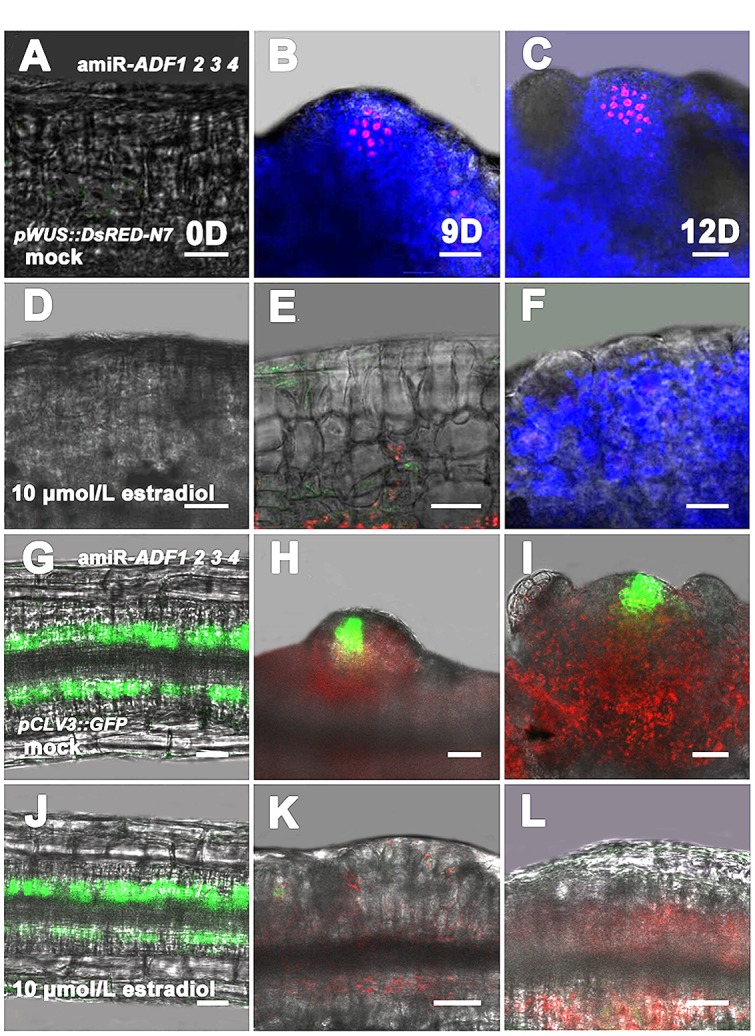
**Expression patterns of *pWUS::DsRED-N7* and *pCLV3::GFP-ER* in callus.** **(A–C)** Expression patterns of *pWUS::DsRED-N7* in callus from non-induced transgenic plants when cultured on SIM without estradiol for 0, 9, 12 days. **(D–F)** Expression patterns of *pWUS::DsRED-N7* in callus from induced transgenic plants when cultured on SIM with 10 μmol/L estradiol for 0, 9, 12 days. The *WUS* signal could not be detected in the callus of estradiol-induced amiR transgenic lines. **(G–I)** Expression patterns of *pCLV3::GFP-ER* in callus from non-induced transgenic plants when cultured on SIM without estradiol for 0, 9, 12 days. **(J–L)** Expression patterns of *pCLV3::GFP-ER* in callus from induced transgenic plants when cultured on SIM with 10 μmol/L estradiol for 0, 9, 12 days. The *CLV3* signal could not be detected in the callus of estradiol-induced amiR transgenic lines. Bars = 20 μm.

Next, *pWUS::DsRED-N7* reporter lines were used to investigate the expression pattern of *WUS* in the amiR-*ADF1 2 3 4* transgenic plants,. Without the induction of artificial microRNA expression by estradiol, the *WUS* signal was regionally distributed after the callus was grown on SIM for 9 days (**Figures 6A,B**). At 12 days, an intense *WUS* signal was detected in the shoot primordia (**Figure 6C**). Nevertheless, the *WUS* signal could not be detected in the callus of estradiol-induced amiR transgenic lines (**Figures 6D–F**), indicating there was no formation of the organizing center cells. Furthermore, stem cell formation was also detected in the shoot regeneration of amiR-*ADF1 2 3 4* plants using *pCLV3::GFP-ER* reporter (**Figures 6G–L**). In contrast to the control without the induction of artificial microRNA expression, expression of *CLV3* was completely repressed in the callus of estradiol-induced amiR lines on SIM, indicating that no stem cell was initiated.

### Inhibition of *ADF*s Disrupts the Auxin Distribution and Polar Transport in Callus

**FIGURE 7:**
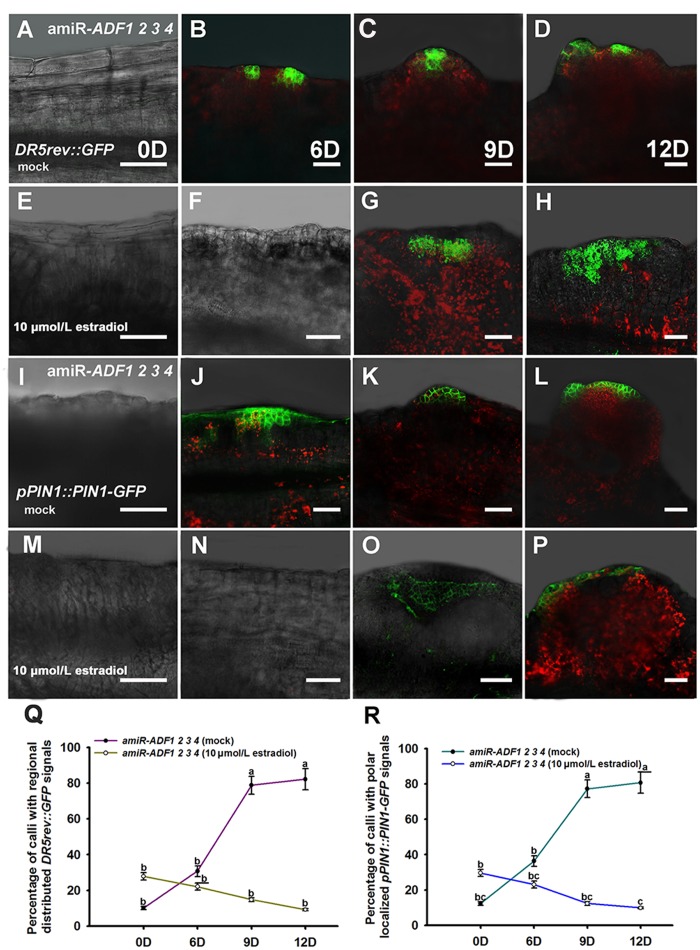
**Auxin distribution and polar transport in callus from amiR-*ADF1 2 3 4* transgenic lines indicated by *DR5rev::GFP* and *pPIN1::PIN1-GFP* expression.** **(A–D)** Expression patterns of *DR5rev::GFP* in callus from non-induced transgenic plants when cultured on SIM without estradiol for 0, 6, 9, and 12 days. **(E–H)** Expression patterns of *DR5rev::GFP* in callus from induced transgenic plants when cultured on SIM with 10 μmol/L estradiol for 0, 6, 9, and 12 days. The *DR5* signal had a dispersed distribution pattern. **(I–L)** Expression patterns of *pPIN1::PIN1-GFP* in callus from non-induced transgenic plants when cultured on SIM without estradiol for 0, 6, 9, and 12 days. **(M–P)** Expression patterns of *pPIN1::PIN1-GFP* in callus from induced transgenic plants when cultured on SIM with 10 μmol/L estradiol for 0, 6, 9, and 12 days. Weak and dispersed signals of *pPIN1::PIN1-GFP* were detected within estradiol–induced callus of amiR-*ADF1 2 3 4* lines. Bars = 50 μm. **(Q)** Percentage of calli with regional distributed *DR5rev::GFP* signals. **(R)** Percentage of calli with polar localized *pPIN1::PIN1-GFP* signals. Different lowercases in **(Q,R)** are significantly different by ANOVA test, *P* < 0.01.

Previously, our study revealed that auxin polar distribution in callus is essential for *Arabidopsis* shoot regeneration (Cheng et al., 2013). To examine whether the establishment of auxin gradients in shoot regeneration was affected by the organization of microfilament cytoskeleton in callus cells, *DR5rev::GFP* signals were examined in the amiR-*ADF1 2 3 4* callus. Spatially restricted distribution of *DR5rev::GFP* signals was firstly identified in some edge regions of non-induced callus on SIM for 6 days (**Figures 7A,B,Q**; **Table 3**). Subsequently, the *DR5* signal gradually became more intense in the promeristem region and extended to the region of the shoot primordia after 12 days on SIM (**Figures 7C,D,Q**; **Table 3**). With the induction of artificial microRNA expression by estradiol, the *DR5* signal was detected after 9 days on SIM but had a dispersed distribution pattern (**Figure 7G,Q**; **Table 3**). Even after 12 days on SIM, little intensive distribution of auxin was observed (**Figure 7H,Q**; **Table 3**).

**Table 3:** Percentage of calli with regional distributed *DR5rev::GFP* signals or polar localized *pPIN1::PIN1-GFP* signals.

	Duration on SIM (day)			
	
	0 day	6 days	9 days	12 days
*DR5rev::GFP*	10.16% (9/87)	30.77% (28/91)	78.82% (67/85)	82.14% (69/84)
*DR5rev::GFP***^∗^**	27.85% (22/79)	22.09% (19/86)	14.80% (12/81)	9.21% (7/76)
*pPIN1::PIN1-GFP*	12.36% (11/89)	36.36% (32/88)	77.22% (61/79)	80.68% (71/88)
*pPIN1::PIN1-GFP***^∗^**	29.63% (24/81)	23.08% (21/91)	12.36% (14/89)	9.88% (8/81)


***^∗^**Induced callus of induced amiR-*ADF1 2 3 4* lines.*

*All the plants are in col ecotypes.*

To determine whether auxin polar transport was disturbed when the *ADF*s were inhibited, the *pPIN1::PIN1-GFP* lines were crossed with the artificial microRNA transgenic plants. Polar localization of PIN1 was clearly observed at edge regions of some non-induced calli for 6 days after transfer of the calli to SIM (**Figures 7I,J,R**; **Table 3**). PIN1 became more polarized in calli for 9 days and 12 days during incubation on SIM without estradiol (**Figures 7K,L,R**; **Table 3**). However, weak and dispersed signals of *pPIN1::PIN1-GFP* were detected within estradiol–induced callus of amiR-*ADF1 2 3 4* lines (**Figures 7M–P,R**; **Table 3**). These results suggested that inhibition of *ADF*s disrupted the auxin distribution and polar transport during *in vitro* shoot regeneration.

## Discussion

Actin filament dynamics are essential for multiple developmental processes in plant cells, especially polarized growth and pattern establishment (Vantard and Blanchoin, 2002). In the process of maize somatic embryogenesis, rearrangement of the cytoskeleton is important for the most critical switch from non-polar to polar units in the embryogenic cells (Samaj et al., 2003), indicating that cytoskeleton organization are essential for the induction of somatic embryogenesis. In animal cells, the cytoskeleton is associated with stem cell specification and the cell division direction (Mammoto and Ingber, 2009; Yima et al., 2009). The cytoskeleton-associated proteins function in both mitosis and cytokinesis in plant cells (Li et al., 2015). For example, Myosin VIII, which is an actin-based molecular motor, plays a role in guiding phragmoplast expansion to the cortical division site by association with both microtubule ends and actin (Wu and Bezanilla, 2014). Moreover, the polarity and axes *de novo* induction during cell wall formation are controlled by dynamic actin in protoplasts (Zaban et al., 2013). Further, in *Arabidopsis* nuclei, actin subclass I variants ACT2 and ACT8 were sub-localized throughout the nucleoplasm while subclass II variant ACT7 was found more concentrated in nuclear speckles, which confirm the existence of actin in plant nucleus (Kandasamy et al., 2010). Auxin polar transport has been identified as a central element of pattern formation. It is interesting that auxin controls its own transport by changing the construction of actin filaments (Maisch and Nick, 2007). In this study, we showed that stem cell formation was associated with the organization of the microfilament cytoskeleton during *in vitro* shoot regeneration. With phalloidin treatment or suppression of *ADFs* which are involved in actin polymerization at the shoot induction stage, normal actin filament depolymerization was inhibited and stem cell formation was disrupted. As the important roles of actin dynamics in mitosis and cytokinesis, polarity induction during cell wall formation and nuclear transport, we inferred that a general impairment of cell growth or division due to actin stabilization might occur with phalloidin treatment or suppression of *ADFs* during *in vitro* formation of the shoot meristem.

Auxin is a multi-functional phytohormone that regulates nearly all aspects of plant growth and development (Teale et al., 2006). Recent studies have shown that auxin modulates cell polarization by activating the ROP GTPase signaling pathway, which directly regulates cytoskeletal dynamics and organization (Lin et al., 2015). Furthermore, actin filaments participate in auxin transport by enabling actin-dependent trafficking of auxin transport components (Muday, 2000; Dhonukshe et al., 2008; Nick et al., 2009). A polarized auxin distribution is also critical for initiation of the shoot primordium during the *de novo* formation of a shoot meristem (Gordon et al., 2007). We showed here that both the auxin polar transport and auxin gradient distribution were disrupted by inhibiting actin depolymerization through phalloidin treatment or simultaneous suppression of *ADF1. ADF2. ADF3*, and *ADF4* expressions, resulting in low frequencies and rates of shoot regeneration. These results suggest that inhibition of microfilament depolymerization disrupts the normal polar transport and distribution of auxin, which are required for the *in vitro* formation of the shoot meristem.

A total of 11 *ADF* genes have been characterized in *Arabidopsis* and are divided into four subclasses (Ruzicka et al., 2007). *ADF1* has been reported to control the actin organization and affect multiple cellular and tissue morphogenesis processes, such as root hair growth, flowering timing and hypocotyl growth (Dong et al., 2001; Gilliland et al., 2002; Bamburg and Bernstein, 2008). *ADF7* is specifically expressed in pollen and regulates actin cable turnover to promote normal pollen tube growth by severing actin filaments (Zheng et al., 2013). Moreover, knockdown of *ADF* genes in the moss *Physcomitrella patens* leads to a star-like radiating distribution of the cytoskeleton and results in the inhibition of tip growth. However, complementation of the *ADF* genes recovers the cytoskeleton distribution to a normal “fringe” structure and enables normal tip growth (Augustine et al., 2008). In this study, we showed that a single *adf* mutation had nearly no effect on shoot regeneration, while simultaneously suppressing *ADF1. ADF2. ADF3*, and *ADF4* using an artificial microRNA method resulted in irregular frequencies and rates of shoot regeneration. These results suggest that *ADF* genes are functionally redundant in regulating microfilament organization and important for *in vitro* formation of the shoot meristem.

## Conclusion

We showed the organization of the microfilament cytoskeleton during *in vitro* shoot regeneration and demonstrated that *ADF*s are critical for actin filament depolymerization, which are required for stem cell formation. Furthermore, the polar transport and distribution of auxin which play important roles during normal shoot organogenesis are involved in actin filament depolymerization. These results provide new molecular insight into the regulation of the organization of the microfilament cytoskeleton in stem cell initiation and *de novo* shoot regeneration.

## Author Contributions

LT and YS designed the research. LT and XL performed the research. YD analyzed the data. YS and XZ wrote the paper.

## Conflict of Interest Statement

The authors declare that the research was conducted in the absence of any commercial or financial relationships that could be construed as a potential conflict of interest.

## Footnotes

1 http://wmd3.weigelworld.org
