# Supplementary material for: Microfilament Depolymerization Is a Pre-requisite for Stem Cell Formation During In vitro Shoot Regeneration in Arabidopsis
Source: Front Plant Sci. 2017 Feb 14;8:158. doi: 10.3389/fpls.2017.00158 (PMC5306138; doi:10.3389/fpls.2017.00158)
Supplement: Supplementary file 1 [file Table_1.DOC]

**Supplementary Table S1 | Primers used in this study.**

| Primer name | Primer sequence (5'-3') | Method |
| --- | --- | --- |
| adf1adf2adf3adf4-I | GATTTAGAGCTCGCATAGATCATTCTCTCTTTTGTATTCC | Artificial microRNA vector cloning |
| adf1adf2adf3adf4-II | GAATGATCTATGCGAGCTCTAAATCAAAGAGAATCAATGA | Artificial microRNA vector cloning |
| adf1adf2adf3adf4-III | GAATAATCTATGCGACCTCTAATTCACAGGTCGTGATATG | Artificial microRNA vector cloning |
| adf1adf2adf3adf4-IV | GAATTAGAGGTCGCATAGATTATTCTACATATATATTCCT | Artificial microRNA vector cloning |
| A | CTGCAAGGCGATTAAGTTGGGTAAC | Artificial microRNA vector cloning |
| B | GCGGATAACAATTTCACACAGGAAACAG | Artificial microRNA vector cloning |
| WUS-F | CCAGCTTCAATAACGGGAATTTAAATCATGCA | qRT-PCR |
| WUS-R | TCATGTAGCCATTAGAAGCATTAACAACACCACAT | qRT-PCR |
| CLV3-F | GTTCAAGGACTTTCCAACCGCAAGATGAT | qRT-PCR |
| CLV3-R | CCTTCTCTGCTTCTCCATTTGCTCCAACC | qRT-PCR |
| ADF1-F | TGCTCTGATATTAGTGTT | qRT-PCR |
| ADF1-R | TTCTCCTCAATCTTGTAA | qRT-PCR |
| ADF2-F | ACTTCATTCTACTTGGTATTATTG | qRT-PCR |
| ADF2-R | AGGAGATGCTTGATATAGGA | qRT-PCR |
| ADF3-F | CTTCTTCGTCTCTTCAAT | qRT-PCR |
| ADF3-R | TTCGTCTTCAATTCCATAA | qRT-PCR |
| ADF4-F | TATTCTAACTTACGAGGACTT | qRT-PCR |
| ADF4-R | TCTTCTGGCAATTCTCTG | qRT-PCR |
| ADF5-F | ATTGCTGGTTAGTGCTTAC | qRT-PCR |
| ADF5-R | GTATAATATCCGTTACTCGTAGG | qRT-PCR |
| ADF6-F | TCATCGCTATGTGGTCTT | qRT-PCR |
| ADF6-R | GCAGTCATTATCAGGAAGTG | qRT-PCR |
| ADF7-F | AGGATGAAGATGGTGTATG | qRT-PCR |
| ADF7-R | ATCAGTGGCTTGTAACTC | qRT-PCR |
| ADF8-F | GATACGCTGTCTACGATT | qRT-PCR |
| ADF8-R | CATCTTACTCCGAACTCTC | qRT-PCR |
| ADF9-F | AAGAACAAGAAGACATCATCT | qRT-PCR |
| ADF9-R | ATCATCCGTCATCCACATA | qRT-PCR |
| ADF10-F | CCACTGCTGAGAATATCC | qRT-PCR |
| ADF10-R | ACACCATCTTCATCCTTAC | qRT-PCR |
| ADF11-F | CAGAGAACAATGGCTAAT | qRT-PCR |
| ADF11-R | TGAACACAATGAACCTATA | qRT-PCR |
| TUB2-F | ATCCGTGAAGAGTACCCAGAT | qRT-PCR |
| TUB2-R | AAGAACCATGCACTCATCAGC | qRT-PCR |
