# Supplementary material for: Microfilament Depolymerization Is a Pre-requisite for Stem Cell Formation During In vitro Shoot Regeneration in Arabidopsis
Source: Front Plant Sci. 2017 Feb 14;8:158. doi: 10.3389/fpls.2017.00158 (PMC5306138; doi:10.3389/fpls.2017.00158)
Supplement: Supplementary file 2 [file Image_1.PDF]

Supplementary Figure S1.

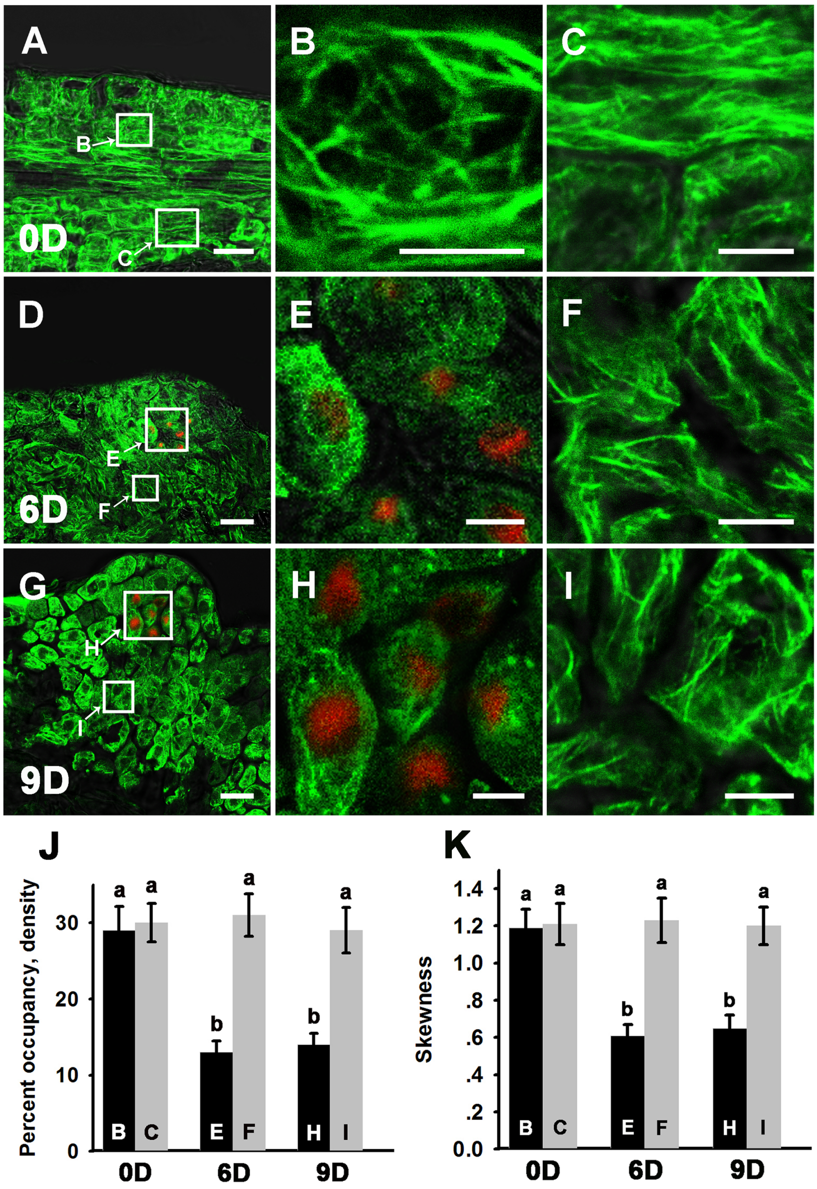

Supplementary Figure S1 | Organization of actin filaments in the callus cells during shoot regeneration using fixation and staining with Alexa-488 phalloidin. **(A)** Callus cultured on SIM for 0 days. **(B,C)** Magnification of the areas indicated by the arrows in (A). Strong green signals of polymerized and bundled filaments were detected both in the epidermal callus cells (B) and in the inner layers of callus cells close to the vascular tissue (C). **(D)** Callus cultured on SIM for 6 days. **(E,F)** Magnification of the areas indicated by the arrows in (D). Microfilaments became more fragmented and less bundled in the *WUS*-expressing cells (E) compared with the callus cells surrounding these *WUS*-expressing cells (F). **(G)** Callus cultured on SIM for 9 days. **(H,I)** Magnification of the areas indicated by the arrows in (G). The green signals of microfilaments mainly showed fragmented distributions in the *WUS*-expressing organizing center cells of the *de novo* shoot meristem (H) compared with the callus cells close to the shoot primordial (I). Green signal represents the fluorescence of phalloidin, red signal represents the fluorescence of

pWUS::DsRED-N7. Bars = 10 μm. **(J)** The average filament density measured in the callus cells shown in (B), (C), (E), (F), (H), (I). The *WUS*-expressing cells had a lower density value than the callus cells. **(K)** The extent of filament bundling (skewness) measured in the callus cells shown in (B), (C), (E), (F), (H), (I). The *WUS*-expressing cells had a lower skewness value than the callus cells. Different lowercases in (J) and (K) are significantly different by ANOVA test, $P < 0.01$. Error bars represent standard deviations from triplicate measurements.
