# Supplementary material for: Microfilament Depolymerization Is a Pre-requisite for Stem Cell Formation During In vitro Shoot Regeneration in Arabidopsis
Source: Front Plant Sci. 2017 Feb 14;8:158. doi: 10.3389/fpls.2017.00158 (PMC5306138; doi:10.3389/fpls.2017.00158)
Supplement: Supplementary file 3 [file Image_2.PDF]

Supplementary Figure S2.

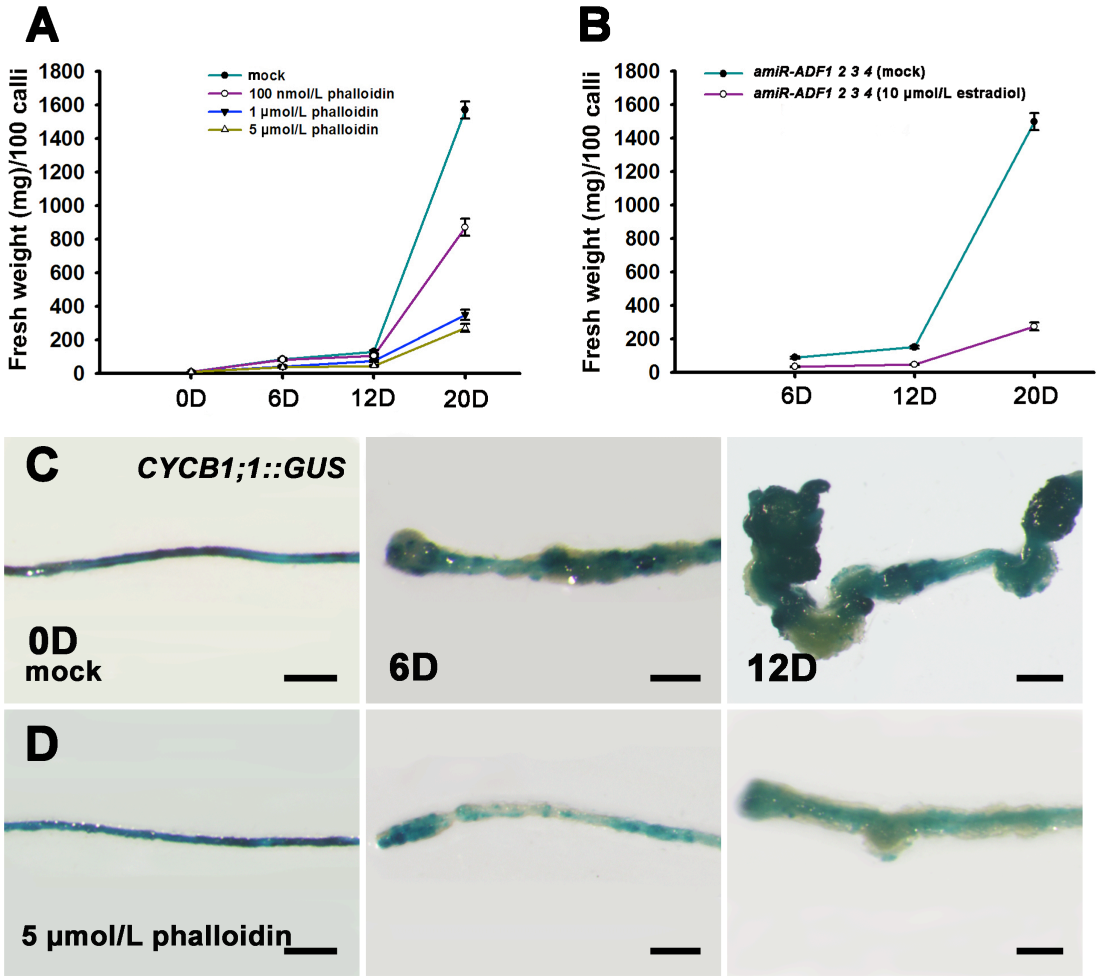

Supplementary Figure S2 | The status of cell growth and mitosis in callus treated with phalloidin.

(A) Fresh weight of callus cultured on SIM with various concentrations of phalloidin for 20 days. **(B)** Fresh weight of callus from amiR-*ADF1 2 3 4* cultured on SIM for 20 days. **(C)** Expression patterns of *CYCB1;1::GUS* in the callus cultured on SIM for 12 days. **(D)** Expression patterns of *CYCB1;1::GUS* in the callus cultured on SIM under treatment of 5 μmol/L phalloidin for 12 days.
